# Understanding tailoring to support the implementation of evidence-based interventions in healthcare: The CUSTOMISE research programme protocol

**DOI:** 10.12688/hrbopenres.13675.1

**Published:** 2023-01-25

**Authors:** Sheena M McHugh, Fiona Riordan, Claire Kerins, Geoff Curran, Cara C Lewis, Justin Presseau, Luke Wolfenden, Byron J Powell

**Affiliations:** 1 School of Public Health, University College Cork, Cork, Ireland; 2 University of Arkansas for Medical Sciences, Little Rock, Arkansas, USA; 3 Kaiser Permanente Washington Health Research Institute, 1730 Minor Avenue, Suite 1600, Seattle, Washington, USA; 4 Clinical Epidemiology, Ottawa Hospital Research Institute, Ottawa, Canada; 5 College of Medicine, Health and Wellbeing, The University of Newcastle, Callaghan, Australia; 6 Division of Infectious Diseases, John T. Milliken Department of Medicine, School of Medicine,, Washington University in St. Louis, St. Louis, Missouri, USA; 7 Center for Mental Health Services Research, Brown School, Washington University in St. Louis, St. Louis, Missouri, USA; 8 Center for Dissemination & Implementation, Institute for Public Health, Washington University in St. Louis, St. Louis, Missouri, USA

**Keywords:** tailoring, implementation strategies

## Abstract

Although there are effective evidence-based interventions (EBIs) to prevent, treat and coordinate care for chronic conditions they may not be adopted widely and when adopted, implementation challenges can limit their impact. Implementation strategies are “methods or techniques used to enhance the adoption, implementation, and sustainment of a clinical program or practice”. There is some evidence to suggest that to be more effective, strategies should be *tailored*; that is, selected and designed to address specific determinants which may influence implementation in a given context.

Despite the growing popularity of tailoring the concept is ill-defined, and the way in which tailoring is applied can vary across studies or lack detail when reported. There has been less focus on the part of tailoring where stakeholders prioritise determinants and select strategies, and the way in which theory, evidence and stakeholders’ perspectives should be combined to make decisions during the process. Typically, tailoring is evaluated based on the effectiveness of the tailored *strategy*, we do not have a clear sense of the mechanisms through which tailoring works, or how to measure the “success” of the tailoring process. We lack an understanding of how stakeholders can be involved effectively in tailoring and the influence of different approaches on the outcome of tailoring.

Our research programme, CUSTOMISE (Comparing and Understanding Tailoring Methods for Implementation Strategies in healthcare) will address some of these outstanding questions and generate evidence on the feasibility, acceptability, and efficiency of different tailoring approaches, and build capacity in implementation science in Ireland, developing and delivering training and supports for, and developing a network of, researchers and implementation practitioners. The evidence generated across the studies conducted as part of CUSTOMISE will bring greater clarity, consistency, coherence, and transparency to tailoring, a key process in implementation science.

## Introduction

Ageing populations and the resultant burden of chronic disease, present significant challenges for health systems worldwide ^1^. Chronic disease management is complex, requiring multidisciplinary healthcare professionals to work in new ways and across organisational boundaries ^2, 3^. Despite effective evidence-based interventions (EBIs) to prevent, treat and coordinate care for chronic conditions ^2^, interventions have struggled to gain widespread adoption, and when adopted, implementation challenges can limit their impact ^4, 5^.

Implementation strategies are “methods or techniques used to enhance the adoption, implementation, and sustainment of a clinical program or practice”. Strategies can often be multi-faceted and involve different processes, for example, planning (conducting a needs assessment), educating (conducting ongoing training), financing (altering incentives), and managing quality (clinician reminders, audit and feedback) ^6, 7^. It is increasingly recommended that implementation strategies are *tailored* ^8– 11^; that is, they are selected and designed to address specific factors which may influence implementation (referred to as determinants) in a given context. 

Despite the growing popularity of tailoring there are gaps in our understanding of the process ^12^. First, the concept of tailoring is ill-defined; while it is commonly considered to be a prospective process to select and modify strategies to address factors which influence implementation, it has also been used to describe modifications or personalisation made in advance to fit with different population subgroups ^13– 15^. In contrast, it has also been used to describe adaptations made *during delivery* of an evidence-based intervention ^10^. Second, the way in which tailoring is applied can vary across studies or lack detail when reported. Broadly speaking, tailoring often involves some or all of the following steps: (a) the identification of implementation determinants, (b) prioritisation of determinants, and (c) selection of strategies by matching to determinants ^16– 19^, but the way in which these steps have been operationalised differs across studies. Third the mechanism by which tailoring is expected to work is unclear ^9^. Typically, tailoring is evaluated based on the effectiveness of the tailored *strategy* to change implementation of recommended practice ^8^. Proximal measures of the effectiveness of the tailoring *process* have yet to be determined (e.g. evaluating strategies prioritised by stakeholders based on empirical evidence to suggest *potential* for impact ^17^). Without a clear sense of the underpinning logic of tailoring, it can be difficult to determine how tailoring can be made more effective and how to measure the “success” of tailoring. **To address these gaps in how tailoring has been conceptualised, operationalised, and evaluated, we will conduct a scoping review** ^20^ **to characterise the processes and outcomes of tailoring.**

Previous work has demonstrated the effectiveness of tailored implementation strategies; a 2015 Cochrane review identified 32 studies and reported a small to moderate effect of tailored strategies on professional practice compared to no strategy or a non-tailored strategy ^8^. They suggested a number of reasons for the moderate effect: lack of fidelity to the tailored strategies; incomplete identification of the determinants; short follow-up period, and; insufficient matching of strategies with determinants ^16^. Since the publication of the last update ^8^, several new studies of tailored strategies have been published ^10, 21– 23^, with dedicated journals established to keep pace with the growing number of implementation research studies following the launch of the flagship journal, Implementation Science. These include Implementation Science Communications (2020), Implementation Research and Practice (2020), and Global Implementation Research and Applications (2021). Consequently, there may be additional evidence on the effectiveness of tailoring or on how it can be undertaken most effectively. Authors of the previous review concluded that methods of tailoring are not yet well developed and are not described in detail in published studies. This may have changed since the last review over seven years ago. **Therefore, we plan to update the existing Cochrane review** ^8^ **to assess the effectiveness of tailored implementation strategies on objectively measured adherence to recommended practice, patient outcomes, or adverse effects.**

While previous work has explored approaches to identify determinants ^16^, there has been less focus on determinant prioritisation and strategy selection. For example, a large multinational study of tailoring, the Tailored Implementation for Chronic Disease (TICD) study, compared different methods to involve stakeholders to identify determinants ^24^, but the latter stages of tailoring were not explored. The process of prioritising determinants and selecting strategies in different settings is not always made clear ^16^. While determinants may be identified as part of a tailoring process, the strategies selected to address them may not necessarily be suitable ^25, 26^. While theory, evidence, and stakeholders’ perspectives are proposed as important elements of the tailoring process ^9^, the way in which these elements should be combined to make decisions during the process is unclear ^27^. More specifically, how different criteria and evidence are used to guide decisions during tailoring is not well understood. Often stakeholder’s decisions about which factors to prioritise and which strategies to select or modify may be based perceptions of feasibility and importance ^17, 19, 26^. In recent years, research has explored policy makers preferences for, and their use of, different types of evidence in decision making ^28– 30^. There have been calls to better understand decision-making processes, the underlying values held by stakeholders, and what influences decision-making in the context of implementation specifically ^31^. **To develop our understanding of the prioritisation and selection step of tailoring, we will conduct a mixed methods study to explore how stakeholders make decisions during these stages of the tailoring process, including what guidance and evidence they use and value.**

There are concerns ^19^ that a divide is growing between best practices from implementation research and what is actually happening and feasible, in practice, for clinical stakeholders. Approaches to tailoring may differ in terms of their feasibility, acceptability, and level of involvement depending on the demands and constraints within a project, organisation, or wider system. Overall, we lack an understanding of how and when to involve stakeholders in tailoring and how different approaches shape the output ^32^. We do not know which approaches to tailoring are feasible and acceptable to stakeholders and in what circumstances and contexts. **We will use a multiple case study approach to compare stakeholders’ experiences of tailoring implementation for different health care priorities and in different health service settings.**

Lastly, there is a growing need for education and training for researchers in the field and practitioners tasked with implementation in the health system. A critical mass of expertise in implementation science (IS) is required now more than ever in Ireland, given the dominant position of implementation “front and centre in achieving reform” in our 10-year national health strategy ^33^. **Through this research programme we will build capacity in IS in Ireland, develop and deliver training and supports for researchers and implementation practitioners, and establish a network of researchers and practitioners across discipline involves in health research.** A training programme, the Irish Implementation Science Training Institute (ISTI) will be developed based on existing examples (e.g., National Institute for Health (NIH) Training Institute in Dissemination and Implementation Research in Health (TIDIRH)) ^34^ We will conduct a longitudinal evaluation of ISTI using an uncontrolled before-after study design and process evaluation. To inform the establishment of the Irish network we will learn from international implementation networks ^35, 36^. 

In the national context, this programme of research is timely given that implementation forms a central pillar of Sláintecare ^33, 37^, the most recent, and first cross-party, national 10-year reform programme, launched in 2017. Sláintecare gives precedence to developing new models of care for frail older adults and those with long-term conditions, based on international best practice, to optimise healthcare delivery, enhance patient experience and improve health outcomes. The reform programme recognizes the need to tailor models of care to population need and allow sufficient flexibility during implementation to respond to local context. Dedicated implementation plans ^33, 37^ set out governance and accountability structures for the reform programme and emphasise the role of stakeholder and public engagement. Focus has shifted from simply introducing service changes, to taking steps to understand how they can be adopted, embedded, and sustained. For example, the Slaintecare Integration Fund ^38^ was dedicated to piloting and scaling best practice examples for chronic disease management and care of older people with explicit focus on learning from the implementation of smaller scale examples across the country. Implementation plans have also begun to be included in national clinical guidelines ^39^, evidence of a growing awareness of the importance of implementation.

### Aims and objectives

Our research programme, CUSTOMISE (Comparing and Understanding Tailoring Methods for Implementation Strategies in healthcare) will address gaps concerning what constitutes tailoring and how it has been applied, and yield insight into the decision-making process during the prioritisation and selection stage. This five-year research programme is funded by the Health Research Board Research Leader Award. Our overarching aim is to identify the core components of tailoring and gather evidence on the feasibility, acceptability, and influence of different tailoring approaches to develop an enhanced understanding of how tailoring works in different service contexts. To achieve our overarching aim, our four goals in the programme are to:

1. Conduct a scoping review to explore how tailoring has been defined, conceptualised, operationalised (timing, level, and scope) and evaluated within the healthcare context, to identify knowledge gaps and future research priorities.
2. Update an existing Cochrane review to assess the effectiveness of tailored implementation strategies on objectively measured adherence to recommended practice, patient outcomes, or adverse effects.
3. Conduct a mixed methods study of a tailoring process to understand decision-making, and approaches to combine the key ingredients of tailoring (i.e., stakeholder engagement, theory, and evidence) within the prioritisation and selection stage. This study will focus on developing tailored strategies to support the national expansion of a structured type 1 diabetes education programme in Ireland. More EBIs may be added as the research programme progresses in response to the policy or practice priorities identified by stakeholders within the health service.
4. Use a multiple case study approach to compare stakeholders’ experiences of tailoring implementation for different health care priorities and in different health service settings.
5. Build capacity in IS in Ireland, developing and delivering training and supports for, and developing a network of, researchers and implementation practitioners.

The goals, design and objectives of our research programme are set out in Table 1.

**Table 1.** Research programme goals, design and objectives.

Goal	Design and objectives
EVIDENCE SYNTHESIS	
** 1. Understand how tailoring** ** has been conceptualised,** ** operationalised, and evaluated** ** in the healthcare context.**	**Design:** Scoping review, including experimental studies and non-experimental studies. **Objectives:** • Explore how tailoring has been defined and conceptualised in the healthcare literature • Examine how tailoring has been operationalised (timing, level, and scope) • Determine how tailoring has been evaluated • Identify knowledge gaps and future research priorities.
** 2. Assess the effectiveness of** ** tailored implementation ** **strategies**	**Design:** Systematic review; update of a Cochrane review ‘Tailored interventions to address determinants of professional practice’ [most recent search conducted in December 2014]. **Primary objectives:** • Determine whether tailored strategies are effective in improving professional practice and healthcare outcomes, answering this question by comparing: - the effectiveness of implementation strategies tailored to address identified determinants of practice compared to *no strategy* - the effectiveness of implementation strategies tailored to address identified determinants of practice compared to *non-tailored strategies* **Secondary objectives** • To assess whether the effects of tailored strategies differ according to whether theory, evidence and stakeholders were involved in the tailoring process • To assess whether the effects of tailored strategies differ according to setting (high or low income)
** 3. Understand decision-making** ** and approaches to combine** ** the key ingredients of tailoring** ** (i.e., stakeholder engagement,** ** theory, and evidence) within** ** the prioritisation and selection ** **stage of the tailoring process.**	**Design:** A series of multisite mixed methods study to tailor implementation strategies to support EBI in the Irish health system. **STUDY 1:** Tailoring strategies to support the implementation of Dose Adjustment for Normal Eating (DAFNE), a group patient education programme for adults with type 1 diabetes **Objectives:** 1. To examine patterns of programme implementation across DAFNE sites (UK and Ireland) (e.g., number of courses delivered, course completion) 2. To understand determinants (barriers and enablers) of implementation in Ireland 3. To work with DAFNE educators in Ireland to develop tailored implementation strategies and examine the criteria used to make decisions during the process
MULTIPLE CASE STUDY	
** 4. Understand stakeholder’s** ** experiences of tailoring and** ** outcomes of the tailoring ** **process to understand how** ** best to support this practice in** ** the health system.**	**Design:** Multiple case study of different tailoring approaches carried out in the individual studies. **Objectives:** • Determine the acceptability, feasibility, and sustainability of different approaches to tailoring and how they influence resulting implementation strategies. • Identify the advantages/disadvantages of different approaches. • Elucidate the underlying mechanism of how tailoring works. • Determine important outcomes for stakeholders at the end of the tailoring process. • Compare approaches using proximal outcomes of tailoring success as identified through the scoping review (Goal 1) which may include types of determinants identified, theoretical coherence of the proposed strategy, evidence of strategy effectiveness. • Determine how much these different approaches cost. • Explore what guidance and evidence stakeholders use and value during prioritisation and selection and in what form.

## Protocol

### Workstreams

**Figure 1.**
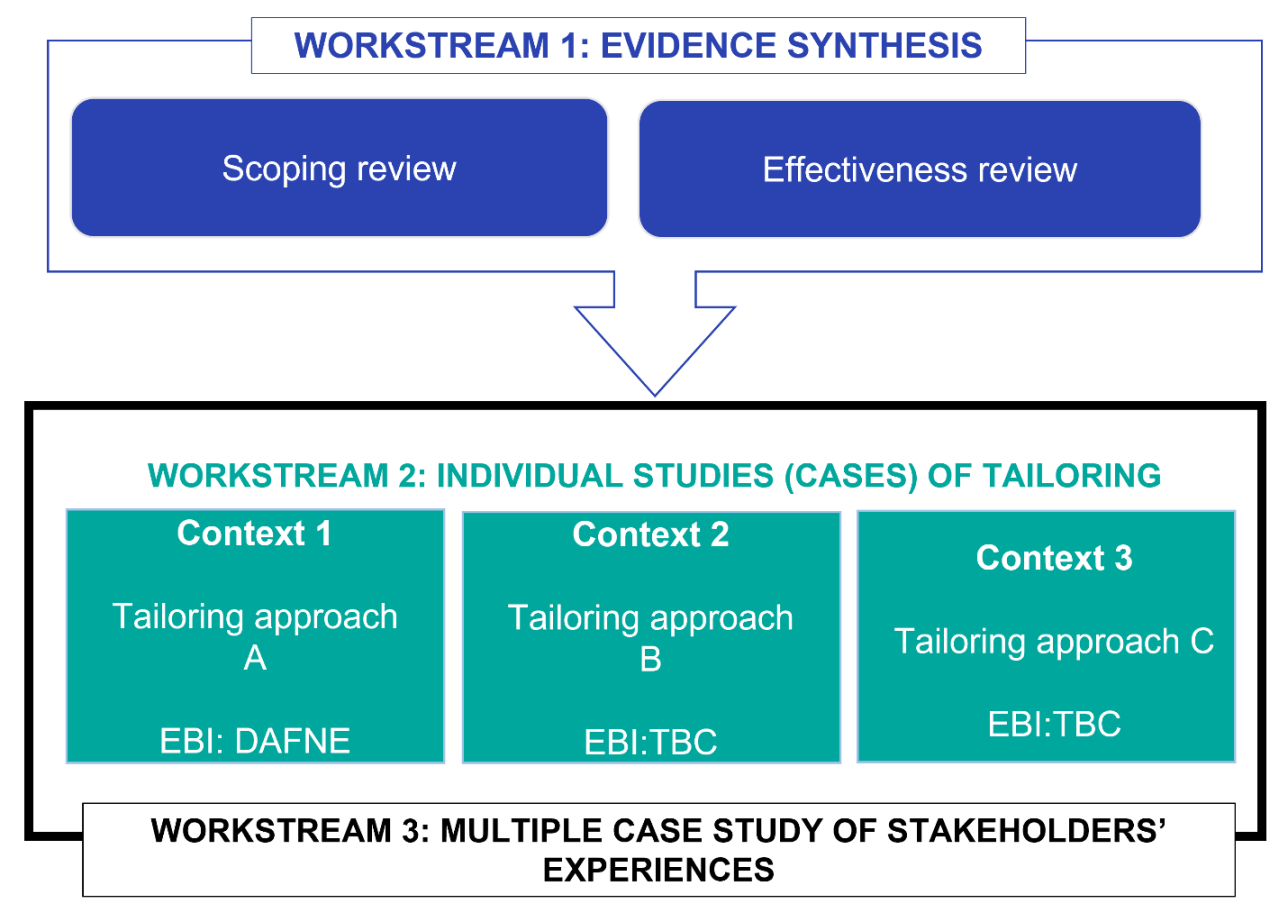
Overview of the three workstreams: evidence synthesis, individual studies to explore the prioritisation and selection stage, and a multiple case study of stakeholder’s experiences of tailoring.

There are currently three work streams. Work stream 1 involves evidence synthesis on how tailoring is conceptualised and operationalised and its effectiveness (Goals 1 and 2). In Work stream 2 we will conduct individual tailoring studies to further our understanding of the prioritisation and selection stage of tailoring (Goal 3). The first study in this work stream will examine decision-making and the use of evidence in the context of tailoring implementation for a structured type 1 diabetes education programme. Alternative tailoring approaches may be used and more EBIs will be added to explore questions arising from the evidence synthesis, including how best to utilise theory and evidence within the prioritisation and selection stage of tailoring. Work stream 3 will be a multiple case study of different tailoring approaches carried out in the individual studies (Goal 4) which will enable us to develop an enhanced understanding of how tailoring works in different service contexts, complementing the findings from the evidence synthesis work. As separate protocol papers on the evidence synthesis workstream are available ^20, 40^ we provide more detail on Workstreams 2 and 3 ( Figure 1).

## Work stream 1: Evidence synthesis

### Scoping review to characterise the processes and outcomes of tailoring

We are conducting a scoping review to identify and organise the existing literature on tailoring implementation strategies in healthcare settings. Scoping reviews are useful to synthesise and categorise existing literature particularly to clarify definitions, concepts and to determine research gaps ^41^.

More detail is available in the published protocol ^20^. In brief, the specific objectives are 1) to determine how tailoring has been defined and conceptualised in healthcare contexts), 2) how it has been operationalised and 3) evaluated. Through seeking to answer the above questions we will determine gaps in the literature) to inform future research on tailoring. To address objective 1 (conceptualisation), we will consider primary studies *and* commentaries or editorials which discuss how tailoring should be done and/or why. To address objectives 2 (operationalisation) and 3 (application), we will only consider primary studies which use tailoring as a proactive process to develop the method of implementation, or modifications of a strategy to fit with different population subgroups (e.g., socio-demographic characteristics; age, gender, culture, socio-economic status) or individual sites. Studies will only be included if they (a) describe the tailoring approach in some detail; the authors describe at least one aspect of the tailoring approach (i.e., when it was conducted, who was involved, format/method used, steps involved, inputs) and (b) at least involve the selection of strategies.

Both qualitative and quantitative primary research studies will be eligible. Unpublished (grey literature) will be included e.g., toolkits, guidance, reports. Only reports and articles in the English language and between the years 2005 and 2022 will be included. Searches have been undertaken across four databases, Medline, EMBASE, Web of Science Core Collection, and Scopus. Handsearching of the journal Implementation Science (2006–2022), BMJ Quality and Safety (2005–2022) and two new journals, Implementation Science Communication (2020, 2021, 2022) and Implementation Research and Practice (2020, 2021, 2022) will be completed. Grey literature will be searched using Google Scholar, using the title-only function, and screening the first 1000 records in accordance with guidance from Haddaway *et al.* ^42^. Full text screening is currently underway.

The review is being conducted in line with the framework and principles promoted by Arksey and O’Malley ^43^ and Levac *et al.* ^44^. The Preferred Reporting Items for Systematic Reviews and Meta-Analyses extension for Scoping Reviews (PRISMA-ScR) guidelines is being followed. 

### Effectiveness review of tailored implementation strategies

The update of the Cochrane review (most recent search completed December 2014) will be conducted in line with available guidance ^45^ to determine the effectiveness of tailored implementation strategies.

Preliminary searches conducted for the scoping review informed changes to the scope and eligibility criteria of the Cochrane Review. More details are included in the review protocol ^40^ but in brief, changes include:

- **Broadening study designs.** We will include all RCT designs, bar crossover RCTs given carry over effects. The previous review focused on cluster RCTs only.
- **Broadening the type of literature.** We will search the grey literature for organisational reports by including two grey literature repositories in the sources to be searched.
- **New terminology.** We will include other terms that have been used to describe tailoring over the last few years (e.g., implementation mapping) or may be considered alternate ways of referring to tailoring which is a collaborative process (i.e., co-design, co-production, co-creation). We have included these terms in the search.

In line with the previous review, we define tailored strategies as “strategies to improve professional practice that are planned, taking account of prospectively identified determinants of practice”. The identification of determinants must have been undertaken before the design and delivery of the strategy. Studies have to involve a comparison group that did not receive a tailored strategy, or a comparison between a strategy that aim to address determinants, compared with a strategy which does not explicitly addressing identified determinants. We will include studies if they assess quality of care, objectively measured adherence of health professionals to recommended practice or guidelines, in a healthcare setting. We will also include studies if they assess patient outcomes, or adverse effects. Patient healthcare outcomes, quality of care, and adverse effects, are as defined in the EPOC guidance on outcomes to be reported in EPOC reviews ^46^.

We have searched six databases; The Cochrane Database of Systematic Reviews (CDSR) and Cochrane Central Register of Controlled Trials (CENTRAL), Medline, EMBASE, Cumulative Index to Nursing and Allied Health Literature (CINAHL) and British Nursing Index (BNI), trial registered and two grey literature sources (Grey Literature Report, Agency for Healthcare Research and Quality). Title and abstract screening is currently underway.

## Work stream 2: Primary studies on the tailoring process

Primary research will be carried out to investigate the role of stakeholders, evidence and theory within the prioritisation and selection stage of tailoring, informed by the findings of the evidence synthesis workstream. This research will involve individual studies to tailor strategies to support the implementation of EBI in the Irish health system, to answer questions about the tailoring process and its core components. Our first study is currently underway. The design of specific studies and the tailoring question they aim to address will be informed by the results of the scoping review.

### Study 1: Tailoring strategies to support the implementation of Dose Adjustment for Normal Eating (DAFNE), a group patient education programme for adults with type 1 diabetes

***DAFNE***

We are conducting a mixed methods study to develop tailored strategies to support the implementation of the structured diabetes education programme for people with type 1 diabetes, Dose Adjustment for Normal Easting (DAFNE) ( Box 1). DAFNE is the only structured education programme that is currently available in Ireland that meets all the criteria of the clinical recommendations regarding structured patient education and thus, has been prioritised for roll-out as part of National Clinical Guideline (Adult type 1 diabetes mellitus) in Ireland ^39^. It is a UK-led programme involving several Irish centres. To become a DAFNE centre, at least one Diabetes Nurse and one Dietitian must train as DAFNE educators, and one physician must train as a DAFNE doctor. Evaluations of DAFNE, both in the UK and Ireland, have focused on effectiveness and psychosocial outcomes ^47– 50^ but little is known about how the programme is implemented across sites and how best to support implementation. Previous studies identified challenges with the implementation of structured diabetes education programmes, including delivery of specific elements of the programme ^51^, and patient non-attendance ^52– 54^.

**Box 1. Overview of Dose Adjustment for Normal Easting (DAFNE)**

- 5-day training course for people with type 1 diabetes
- Groups of 6–8 participants are given guidance by two certified educators (one diabetes specialist nurse and one diabetes dietitian), allowing participants to learn by experience and practice the key skills of estimating carbohydrate and adjusting insulin dose.
- A two to three hour group follow-up session is offered to participants at 4–12 weeks after each course to consolidate the skills acquired during the course and to review targets and goals.
- In response to COVID-19, some centres offered remote DAFNE, which is a blend of online learning, workbook activities and remote group sessions facilitated by DAFNE educators.
- Since COVID-19, more DAFNE Central administrators have started offering the DAFNE ‘buddy’ system which involves a call twice a year to offer support, for example, with entering data, training etc. Any issues are fed back to either clinical educators / trainers or operational staff. DAFNE training and delivery has also moved online.

There are potentially 20 DAFNE sites eligible for this study at different phases of implementation ^55^. At the time of writing, eleven DAFNE centres are already established in Ireland (sustainment). Two centres started delivering courses in 2022 (early implementation), and seven have expressed an interest in or are in the process of joining the DAFNE consortium and expect to be established in 2022 (preparation). As the centres are at different stages of implementation, we have an opportunity to explore tailoring both as a process to identify anticipated determinants and select new tailored strategies to support implementation (early sites), and potentially modify existing strategies to improve and sustain a programme (at established sites). We also have an opportunity to examine readiness for implementation at newer sites, evaluating this before and after the tailoring process.

### Objectives

Our specific objectives for this study are:

1. To examine patterns of programme implementation across DAFNE sites (UK and Ireland)
2. To understand determinants (barriers and enablers) of implementation in Ireland

3. To work with DAFNE educators in Ireland to develop tailored implementation strategies to support the implementation of DAFNE and further our understanding of decision-making during the prioritisation and selection stage, specifically:
  ▪ How do stakeholders make decisions about what to prioritise and what to select?
  ▪ What additional guidance and evidence do they use and value during the prioritisation and selection?

4. To evaluate the feasibility and outcomes of the tailoring process (to understand how best to support this practice in the health system).

### Design and methods

**Figure 2.**
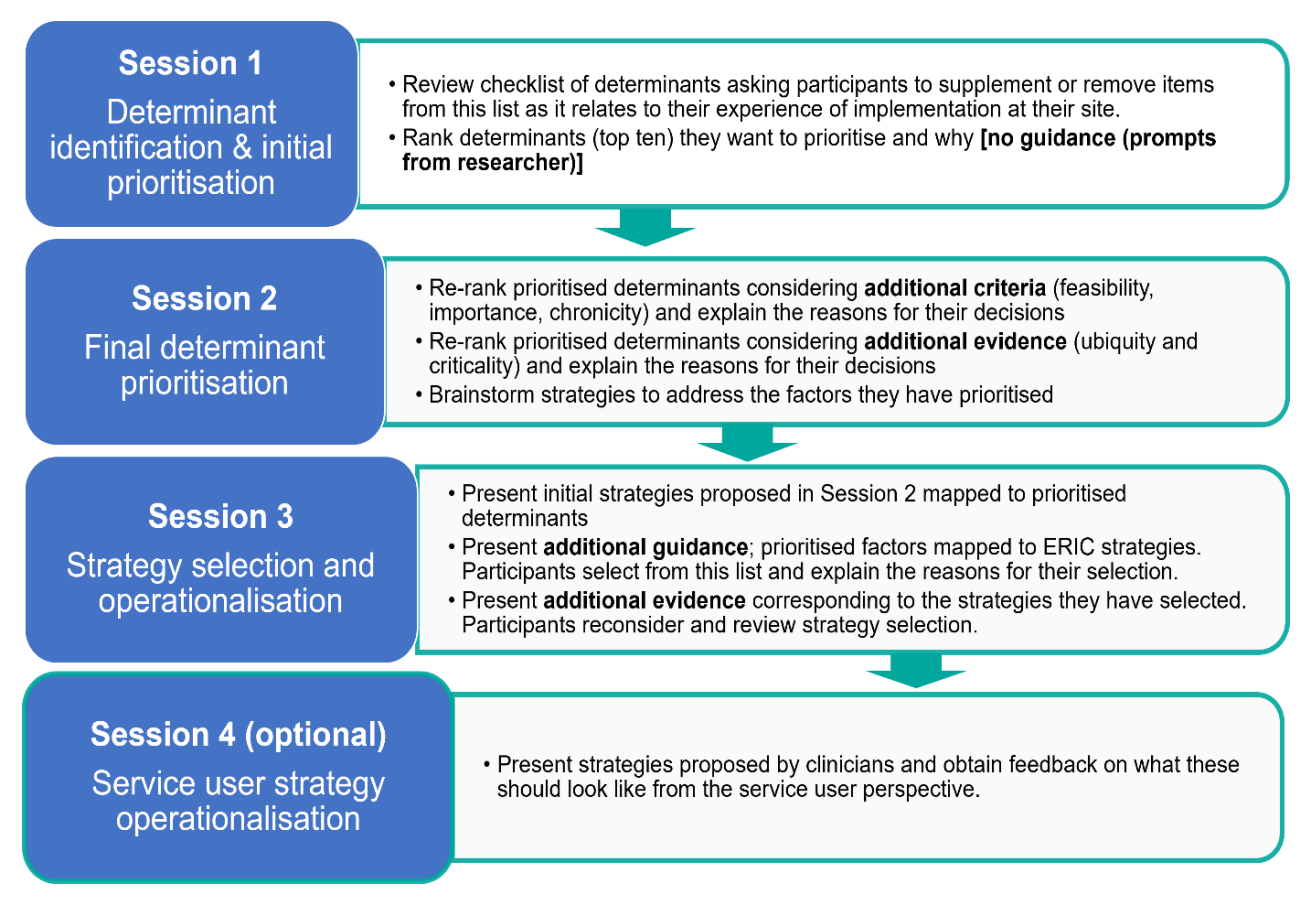
Overview of DAFNE tailoring sessions.

We are conducting a mixed methods study of a structured group consensus-based process. All Irish centres will be invited to take part in three group sessions to work through the steps of the tailoring process: 1. determinant identification, 2. determinant prioritization and 3. selection of strategies, varying the guidance and evidence provided at each step ( Figure 2). Where appropriate, the selected strategies will be applied and evaluated in a future study.

### Recruitment

***Health professionals***

Participants invited to take part in the tailoring process include professionals who are currently involved in or due to be involved in the delivery of DAFNE at each centre (dieticians, diabetes nurse specialists, consultant physicians, and administrators). We anticipate a minimum of three participants to be included at each site.

Centres are being invited to take part in the study via email or phone by hospital site lead clinical investigators and co-investigators from the National Clinical Programme for Diabetes. Teams are provided with an overview of the study and advised that they are under no obligation to take part.

***Service users***

Service users may be invited to participate in a session to discuss and operationalise strategies if patient-level strategies are proposed by centres. Service users will include those who have already attended a DAFNE course or are eligible (and have been invited) to attend the programme. Service users will be adults aged 18 or over, with type 1 diabetes, representing a mix of ages, and genders. Multiple discussion sessions will be held as centres progress through the tailoring process and as strategies are proposed. Service users will have the option to take part in more than one discussion session if they wish. It is anticipated 4–5 service users will be invited to take part in each discussion session.

Service users will be invited via HCPs delivering the DAFNE programme. People who are signed up to attend DAFNE or have recently attended the programme will have the option to contact researchers who will then follow up with them to confirm their interest. Service users will be purposively sampled from respondents who contact the research team. If it is not possible to recruit service users through DAFNE centres, other recruitment avenues will be used: social media posts, Diabetes Ireland clinics and support groups, and information distributed at local and community events. 

All professionals and service users are invited to read a participant information sheet and provide explicit consent to participate in the study.

To date, eight centres are actively participating in the tailoring process. Ethical approval has been granted for six further centres. Ethical approval for five other centres is pending.

### Data collection

***Quantitative data on implementation***

To understand delivery of the DAFNE programmes at different DAFNE centres in the UK and Ireland, we are accessing and analysing (1) anonymised service-level data and participant-level data held by the Central DAFNE Coordinating Centre (1 ^st^ January 2019 – 1 ^st^ January 2021) *and* (2) service-level data held by local centres [one-year pre-COVID-19 (2019) and the most recent years of the programme (2020, 2021, 2022)].

**Central DAFNE data**

Irish centres submit data on course delivery and participants on an annual basis. Where feasible, these data are being used to identify aspects of implementation that may need to be improved. The following anonymized data from Central DAFNE is being accessed to understand the profile of participants and programme delivery:

- Year of birth, sex, ethnicity, year dx, height, weight, HbA1c, number of severe hypoglycaemia episodes and diabetic ketoacidosis (DKAs) of programme participants pre and post course attendance.
- Number of DAFNE courses delivered, number of attendees, dropouts / course completion (number of days completed), reasons for dropouts, curriculum delivered.

**Local data**

The following anonymized data from local sites is being accessed, where feasible, to understand the profile of participants and programme delivery: Number of people eligible, number of people invited, and number who accepted course.

### Tailoring process

Each step involves a 1-hour group discussion session, conducted online or face-to-face depending on what is most feasible for participants. Sessions are being facilitated by FR, a postdoctoral researcher with a PhD in Health Services Research, and experience of conducting qualitative research with health care professionals and patients. FR does not have a prior relationship with participants. Professionals involved in DAFNE delivery and service users (who have been invited to or attended DAFNE) attend separate sessions. The sessions were piloted with a group of doctoral students who are also clinicians and known to the study authors.

***Session 1: Determinant (barrier and enabler) identification and initial prioritisation***

Prior to the first session, participants are asked to complete a survey to describe the characteristics of their DAFNE site. This survey includes validated scales ^56, 57^ to assess inner setting measures as per the Consolidated Framework for Implementation Research (CFIR) (culture, implementation climate, learning climate, leadership engagement, available resources) and readiness for implementation ^58^. Newly established centres at the early stages of implementation will be asked about their readiness for implementation. Data are analysed to understand their unique contexts (e.g., resources, staffing, experience with implementation) and the factors that potentially influence the tailoring process and its feasibility in the health service. As part of this survey, to streamline determinant identification ^59^, they are asked to consider a list of determinants, indicate whether each determinant is a barrier or enabler at their site, and to supplement or remove determinants from this list as they relate to their experience of implementation. This list is based on rapid evidence review of the existing literature on the implementation of structured diabetes education programmes. 

During the first session, discussions are semi-structured and guided by a topic guide to further consider determinants at their site. Participants are asked to initially prioritise their top ten determinants based on their own assumptions individually using an interactive ranking tool, called Slido ^60^. They are asked to discuss their reasons for prioritisation.

***Session 2: Final determinant prioritisation with guidance and evidence***

During the second session, participants are asked to consider the prioritised determinants taking into account specific criteria: the feasibility of addressing a determinant, importance, and chronicity [how frequently the determinant arises in practice or persists over time]. Where available, the research team present additional evidence about ubiquity [how pervasive a determinant is, operationalised as the extent to which a determinant comes up in the international literature and across other Irish centres], and criticality [how likely is the determinant to affect the implementation outcome, operationalised as whether other studies cite the determinant as influential]. Lastly, participants are asked to initially brainstorm what strategies might address the determinants they have prioritised. By exploring the use of additional criteria (chronicity, ubiquity, criticality) we are building on research conducted by the Optimising Implementation in Cancer Control (OPTICC) centre in the US, which proposed these criteria as a way to overcome the limitations of typical criteria used to prioritise determinants (feasibility and importance) which are not clearly linked to impact ^61^.

***Session 3: Strategy selection***

During the final session, when selecting strategies, participants are asked to consider evidence of effectiveness and alignment with prioritized determinants based on existing guidance. Participants are presented first, with strategies they proposed in Session 2 mapped to determinants. If there are strategies which were not mapped to specific determinant(s), participants are prompted to explain how they expect the strategy would work and what it would target.

Second, they are presented with prioritised factors mapped to the Expert Recommendations for Implementing Change (ERIC) strategies ^7^ highlighting the % agreement based on expert consensus ^62^ and acknowledging the limitations of this matching. Participants are asked to select strategies from this list and discuss the reasons for their selection. If available, participants are presented with (systematic review) evidence corresponding to the strategies they have selected. If evidence of strategy effectiveness is unavailable, they are prompted to indicate whether additional evidence (and what type of evidence) would influence their decisions. Lastly, they are asked to select two strategies to focus on and these are further operationalised in the session, prompting them to specify Actor, Action Target, Temporarily, Dose, Outcome and Justification ^63^. If patient-level strategies are selected, in a separate session service users are asked to provide feedback on these strategies and what they should look like (how they should be operationalised) from their perspective.

### Data analysis

***Quantitative analysis***

Anonymised data obtained from DAFNE Central will be analysed using Stata. Descriptive statistics will be generated (mean (sd), median (range), frequencies, percentages) to understand the demographic and clinical profile of people attending DAFNE, compare DAFNE participant profiles pre and post course attendance, and understand course delivery across centres. Variables such as centre and country will be examined as predictors of course completion using logistic regression.

Survey data will be inputted (if paper-based) or downloaded (if online) to Excel for quality checks and cleaning before being imported into Stata for analysis. Survey data will be analysed descriptively (frequencies and percentages). For measures of the inner setting (culture, learning climate, available resources, leadership engagement), mean scores will be calculated across respondents at each site. For the organisational readiness measure, mean scores will be calculated at a subscale level (change commitment and change efficacy) and at the overall readiness level (i.e., combined change commitment and change efficacy).

***Qualitative analysis***

Qualitative analysis will be conducted with the support of NVivo software for data management. Transcripts will be coded using the Framework Method ^64^, which is a form of thematic analysis. This approach is suitable for projects with prespecified objectives such as evaluating acceptability and feasibility. It also allows unexpected themes to emerge during initial phases of familiarisation and open coding.

### Piloting and evaluating tailored strategies

Within each case study, following selection, strategies will be applied and piloted using the most appropriate and feasible methodology ^65^. Depending on whether there are limited sites (as in the DAFNE study) or opportunities to randomise sites to different conditions, then interrupted time-series or controlled before and after comparisons may be feasible. Challenges to evaluating strategies will include the interactive effects of multilevel strategies ^66^ and distinguishing the impact of the tailoring *process* on strategy success from the impact of how the strategy is ultimately deployed. Research suggests that factors relating to the context in which the interventions or strategies are delivered and in particular *how* they are delivered can also be decisive ^67– 69^. For example, length of follow up, the number of intervention contacts, the type of participant population, and demographics, may moderate the effect of tailored interventions ^70^. The evidence synthesis work (scoping review and effectiveness review) may identify important contextual factors to monitor and assess as part of evaluation. If time and resources allow, we will proceed to evaluation potentially utilising more complex designs; for example, if the selected strategy should be individualised (dose, type, delivery) and repeatedly adapted to meet the needs of participants, then a Sequential multiple assignment randomised trial (SMART) design, may be appropriate ^71^.

### Multiple case study to understand stakeholder experiences of tailoring

***Design***

**Figure 3.**
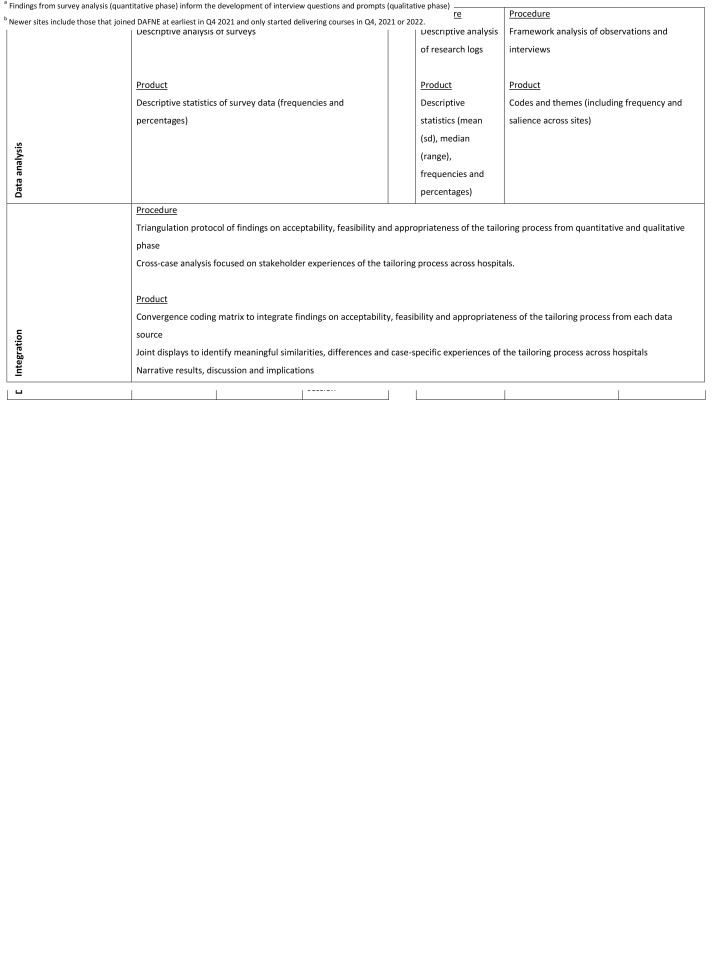
Study design for DAFNE tailoring evaluation.

The design of the multiple case study (Goal 4) will be refined based on the outputs of the evidence synthesis work (Goals 1 and 2) and DAFNE study (Goal 3). A mixed methods convergent design ( Figure 3) will be used to evaluate participants’ experiences of the tailoring process in each case using research logs, non-participant observation of tailoring sessions, follow-up surveys, and post-tailoring interviews with stakeholders who participated in the tailoring process in different studies and service contexts. The purpose will be to assess acceptability (e.g., format, participant mix), appropriateness (e.g., fit for them, their service context/background/role), feasibility (e.g., time taken/commitment required, skills and knowledge required, cost to them), sustainability (e.g., is this a process they could engage in again, supports needed), and suggestions for improvement. An independent researcher not involved in the tailoring process will lead collection and analysis of data gathered from participants for the purposes of *evaluating* the process. This is to ensure participants feel open to give honest feedback on the tailoring process and to ensure data analysis is objective and credible. In terms of methods, the quantitative and qualitative phases will be connected when findings from survey analysis (quantitative phase) inform the development of interview questions and prompts (qualitative phase) ^72^. The quantitative and qualitative results will be integrated during the interpretation of the primary outcome, stakeholder experiences of the tailoring process. The DAFNE study will be the first case. An overview of the purpose and nature of the data collected is provided in Figure 3.

### Quantitative phase

***Surveys***

A survey to assess organisational culture and climate and readiness for implementation will be administered prior to the first tailoring session. This survey consists of validated scales developed by Fernandez and colleagues ^57^. To assess the acceptability, feasibility, and appropriateness of the tailoring process, a survey will be disseminated after each session. This survey is based on the measures developed by Weiner *et al* ^73^. A survey to assess readiness for implementation will be administered to newer centres prior to and upon completion of the tailoring process. This survey consists of a validated scale developed by Shea *et al* ^56^. Participants will be asked about their preferred mode of survey delivery i.e., online or paper based.

### Qualitative phase

***Research logs***

Data collected in research logs will include: the time required for administration, reminders, confirm attendance and schedule sessions, barriers and facilitators to recruitment perceived by researchers, participants’ attendance, and retention across each stage, time taken to analyse group meetings/existing datasets, as applicable, and any additional costs to participants.

***Non-participant observations***

Non-participant observations will be carried out during the tailoring sessions by the independent researcher, recording the following information: purpose of meeting, duration, number and type of participants, nature of dynamics/interactions between participants, decision-making process and criteria used to make decisions, and facilitation of the group by the researcher (including direction and guidance). Any consumables required will also be logged (e.g., printed materials, software). Non-participant observations will also be carried out during tailoring sessions with service users if conducted. Observations will be overt, in that participants will be aware that each tailoring session is being observed.

***Semi-structured interviews***

All participants (clinicians and services users) in the tailoring process will be invited to participate in an interview with the researcher in person, by phone, or online. Results of the non-participant observations and surveys will inform the topic guides which will broadly aim to explore the following aspects of the process:

- Understanding of the tailoring process
- Decision making processes and influences
- Success in identifying the key barriers or facilitators and selecting the ‘best’ strategy from stakeholders’ perspective
- Preferred output and perceptions of success from the tailoring process e.g., action plan, summary, list
- Experience of tailoring including perceptions of acceptability (e.g., format, technical, participant mix, input into discussion making process), appropriateness (e.g., fit for them, their service context/background/role), feasibility (e.g., time taken/commitment required, skills & knowledge required, cost to them), sustainability (e.g., is this a process they could engage in again, supports needed).
- Perceived influence of tailoring on implementation (i.e., elucidate the underlying mechanism of how tailoring works)

A de-brief will be conducted with researchers by the independent observer at the end of the three tailoring sessions for each site and at the end of the tailoring process for each case. It will record the researcher’s impressions of the sessions, how participants worked together, what worked well, and what could have been done differently.

### Data analysis

Quantitative and qualitative analysis of collected data will be performed. Findings from both phases will then be integrated using a triangulation protocol. The analysis will be performed at two levels: within each case and then across the cases ^74, 75^.

***Quantitative analysis***

Survey data will be downloaded to Excel for quality checks and cleaning before being imported into Stata for analysis. Survey data will be analysed descriptively (frequencies and percentages).

***Qualitative analysis***

Qualitative analysis will be conducted with the support of NVivo software for data management. Transcripts will be coded using the Framework Method ^64^, which is a form of thematic analysis. This approach is suitable for projects with prespecified objectives such as evaluating acceptability and feasibility. It also allows unexpected themes to emerge during initial phases of familiarisation and open coding.

***Integration***

A triangulation protocol will be used to integrate findings on acceptability, feasibility, and appropriateness of the tailoring process from the quantitative and qualitative phase ^76^. This will involve creating a “convergence coding matrix” to compare the findings across each source. The relationships between data will be categorised as: (1) silence (only one data source out of the two being compared contained data on a particular finding), (2) dissonance (conflicting findings in the data), (3) partial agreement (complementarity between data) or (4) agreement (convergence in the data) ^77^

For the cross-case analysis, data will be analysed for each case first and then findings compared across cases using matrices (displaying results in rows and cases in columns). Quantitative and qualitative data will be integrated using joint displays to identify meaningful similarities, differences, and case-specific experiences ^72^.

### Patient and public involvement in CUSTOMISE

A panel of PPI partners has been established, comprising 3 women and 2 men with diabetes. Three meetings have been held to date. There is an ongoing challenge of when and how best to include PPI in implementation research given the aim is often to change health professional as opposed to patient behaviours, with a risk of tokenism if patients and members of the public are expected to provide input on aspects with which they have little direct experience (e.g., understanding how health professionals might respond to an audit and feedback report) ^78^. Therefore, we are cognisant of convening the panel to discuss items of most relevance. Their role/remit will potentially change as the project progresses. To date, the panel have advised on the project name (CUSTOMISE), logo and branding, avenues for PPI recruitment, and how to explain the overarching plan for the project to a lay audience. They also advised on questions for the scoping review, along with the format and language used in patient information materials for the DAFNE study. The panel will be refreshed as the project progresses, with targeted campaigns to recruit panel diverse members with experience relevant to the study in question. Throughout the project, the panel will be involved in informing the dissemination strategy, and shaping messages for target audiences, which are sensitive to societal priorities and national policy.

## Discussion

The overarching aim of the programme is to accelerate and enhance the implementation of evidence into policy and practice. This will be achieved through dedicated research on the process and impact of tailoring strategies to improve the implementation of evidence-based interventions for older people and those with chronic conditions. Through evidence synthesis and mixed methods multiple case studies, the programme will build knowledge about tailoring implementation strategies, understanding what is feasible and effective for different stakeholders. This is the first dedicated research programme focused on tailoring methods for implementation strategies. The programme is intended to be flexible and responsive, using the findings from the scoping review as a guide we will design studies to address some of the evidence gaps with respect to tailoring.

### Implementation research in the Irish context: building capacity

As mentioned, implementation forms a central pillar of Sláintecare ^33, 37^. Though the importance of implementation research is growing, changes to health services in Ireland are still often introduced without a systematic approach to developing and applying strategies to specifically support implementation of these changes in the given healthcare context. This may reflect gaps in our understanding about how “best” to tailor strategies from practitioner and researcher perspectives. New services and reform programmes ^79^ continue to face challenges of adoption ^80– 82^, and implementation ^82, 83^. There is a growing need for education and training for researchers in the field and practitioners tasked with implementation in the health system. Our research programme will be undertaken alongside steps to build capacity and training for implementation research in Ireland. Activities will include: (1) the establishment of a network for implementation researchers, and (2) the development and delivery of training and supports for implementation researchers and practitioners. Our training programme in IS, the Irish Implementation Science Training Institute (ISTI) ^84^ open to PhD students, researchers, health professionals and health service management undertaking implementation research or evaluation across health care, public health, and community settings, will run twice over the course of the 5-year project. Informed by other examples of training in dissemination and implementation science in the US and Europe ^85^, ISTI aims to develop beginner and intermediate competencies in IS through a blended format; participants complete online modules followed by in-person workshops and talks. Aligned with many international offerings, ISTI offers participants the opportunity to engage with national and international faculty and to receive advice and guidance from them on their own IS project proposal. In developing the Irish network, we will draw on other international examples, including the Swiss Implementation Science Network (IMPACT) ^35, 36^, which, for example, hosts knowledge exchange events and seminars leveraging national and international expertise to benefit the community of implementation researchers and practitioners. Our capacity-building work also aligns with other national initiatives, including established implementation research groups (Health Implementation Science and Technology cluster ^86^, Co-Lead ^87^) and non-profit organisations such as the Centre for Effective Services ^88^ which is dedicated to supporting implementation in Irish services.

### Research team, collaborators, and health service partners

Collaborators on the programme include international experts in implementation research, and partners working within the health service. The programme in particular is strengthened by strong links to clinical partners, including the National Clinical Programme for Diabetes, National Integrated Care Programme for Older People and Sláintécare Programme Office. The latter will recommend and support evidence-based approaches to implementation across healthcare settings. This partnership with the first dedicated national office for implementation in Ireland will allow for the spread of implementation methods and approaches generated by the research programme and provides long-term potential to compare the effects of different implementation strategies at scale in the health system. Working with health partners will ensure the research has a direct and continuous impact on real-world efforts to disseminate and implement evidence in the health system. Collaborators on the programme include international experts in implementation research (JP, GMC, LW, CCL, BJP).

## Ethical considerations

Ethical approval will be sought from relevant ethics committees including the Social Research Ethics Committee (REC) UCC and Clinical REC of the Cork University Teaching Hospitals. For the DAFNE study, ethical approval is being sought from individual hospital RECs across Ireland. The need to apply separately to each hospital REC is indicative of the challenges with obtaining ethical approval for this type of national study and the lack of appropriate REC structures in Ireland. While a national REC has been established for clinical trials and medical devices ^89^ there is currently no national REC for health services/implementation research of the type outlined in this protocol; that is, relatively low risk studies which, while involving clinicians and service users, are observational and exploratory in nature and do not involve clinical intervention. Given the barriers to this type of research and subsequent delays, costs for the research programme, along with adding burden to hospital REC, efforts to consolidate a REC within the HSE is a welcome step.

## Conclusions

The findings of the CUSTOMISE project will be relevant to both implementation researchers and practitioners. Tailoring is becoming more common, but we lack clarity on how it is conceptualised, operationalised, and evaluated. Findings from our evidence synthesis will be of value to researchers in the field, through describing how tailoring has been undertaken within the healthcare context, identifying research gaps, and informing future priorities. We hope this will prompt the implementation science community to undertake new research to specifically explore and address these gaps. As part of CUSTOMISE we will specifically seek to understand the prioritisation and selection stages of tailoring, the role of theory, evidence, and stakeholders, including stakeholders’ experiences of tailoring for different health care priorities and in different health service settings. By enhancing our understanding of what approaches to tailoring are feasible and acceptable to stakeholders and in what circumstances and contexts, we hope that CUSTOMISE will aid the future application of tailoring to support the uptake and implementation of EBIs in the Irish health system.

## Data Availability

No data are associated with this article. Zenodo: Organisational context survey, https://doi.org/10.5281/zenodo.7468061 ^90^. This project contains the following extended data: -   Organisational context survey_version 1.pdf -   Organisational context survey_version 2.pdf Zenodo: Understanding tailoring to support the implementation of evidence-based interventions in healthcare: The CUSTOMISE research programme protocol - COREQ checklist, https://doi.org/10.5281/zenodo.7468883 ^91^. Data are available under the terms of the Creative Commons Attribution 4.0 International license (CC-BY 4.0).
