# Supplementary material for: Understanding tailoring to support the implementation of evidence-based interventions in healthcare: The CUSTOMISE research programme protocol
Source: HRB Open Res. 2023 Jan 25;6:7. [Version 1] doi: 10.12688/hrbopenres.13675.1 (PMC10285335; doi:10.12688/hrbopenres.13675.1)
Supplement: Peer review history

# Reviewer report: Reviewer response for version 1

**DOI:** 10.21956/hrbopenres.14954.r34073

**Published:** 2024-03-06

**Authors:** Louise Hull

**Affiliations:** 1 King’s College London, London, England, UK

Thank you for the opportunity to review this manuscript. This exciting programme of research is of high interest and importance to implementation researchers and healthcare practitioners responsible for the implementation/delivery of evidence-based interventions. Despite recommendations from experts that implementation strategies should be selected and tailored to address implementation determinants in a given context, very little guidance is available to support implementation researchers and practitioners to do this. This ambitious programme of research aims to address this knowledge/research gap. I have made several specific comments, and raised questions, that I hope the authors will find useful.

Work stream 2

**Objective 3: To work with DAFNE educators in Ireland to develop tailored implementation strategies and examine the criteria used to make decisions during the process.** I would like to understand the rationale for only ‘involving professionals who are currently involved in or due to be involved in the delivery of DAFNE’. Would it not be beneficial to involve all relevant stakeholder groups in the process of tailoring implementation strategies? Although the authors state that ‘service users’ may be invited to participate, it appears their involvement is solely dependent on whether ‘patient-level strategies are proposed by centres’. It would be useful to discuss the rationale and associated limitations of not involving other relevant stakeholder groups.**

**Tailoring process - Each step involves a 1-hour group discussion.** I have serious concerns that 3 hours (3 x 1-hour sessions) is sufficient to identify determinants, prioritise determinants, and select strategies. I suggest the authors justify this approach and highlight the associated limitations.

**

**Session 1: Determinant (barrier and enabler) identification and initial prioritisation. **To improve transparency, it would be useful to provide more detail about the ‘list of determinants’ that participants will use to identify determinants. How many determinants are on the list, is the list based on a particular determinant framework etc.? The authors state that participants will be asked to initially prioritise their top ten determinants - why 10?

**Session 2: Final determinant prioritisation with guidance and evidence. **The expert input described will undoubtedly have a significant impact on the tailoring process/outcome but is not something that all researchers and practitioners will have access to outside of the research context. Will this not limit the generalisability of the findings? Again, I suggest the authors explicitly state and discuss the limitations of this approach.

**Session 3: Strategy selection.** The authors state the participants will be ‘asked to select two strategies to focus on and these are further operationalised in the session, prompting them to specify Actor, Action Target, Temporarily, Dose, Outcome and Justification’. Why only 2? This seems very limited. Are participants expected/encouraged to go through this process for all selected strategies after the last session?

**Piloting and evaluating tailored strategies:**

 It’s unclear how the tailored implementation strategy will be monitored and evaluated throughout implementation. A structured approach to documenting/monitoring adaptations and modifications and the evaluation of implementation strategies seems to be missing.  

**Discussion:**

 Related to the comments and questions raised above, the authors need to explicitly state and discuss the limitations of the research programme.

 Thank you again for the opportunity to review this manuscript - I look forward to learning from the findings of this programme of work.

Is the study design appropriate for the research question?

Yes

Is the rationale for, and objectives of, the study clearly described?

Yes

Are sufficient details of the methods provided to allow replication by others?

Yes

Are the datasets clearly presented in a useable and accessible format?

Not applicable

Reviewer Expertise:

Implementation science, Patient safety

I confirm that I have read this submission and believe that I have an appropriate level of expertise to confirm that it is of an acceptable scientific standard.

# Reviewer report: Reviewer response for version 1

**DOI:** 10.21956/hrbopenres.14954.r34070

**Published:** 2023-06-15

**Authors:** Lea Sacca

**Affiliations:** 1 Florida Atlantic University, Boca Raton, Florida, USA

This article is of high interest to researchers and experts in the dissemination and implementation science field. However, some minor changes are needed to ensure the flow of the content:

1. The introduction is well-written and is supported by sufficient number of references. I would prefer the compilation of the sentences highlighted in bold at the end of the introduction to highlight the purpose of the paper and the written protocol. Going back and forth between background/objectives can make it confusing to the reader to keep track about where they are in the paper.
2. It might be interesting to include a section on expected challenges throughout the implementation of the three workstreams in the discussion section.
3. How will you be ensuring inter-rater reliability when matching ERIC strategies to the determinant-specific strategies? 

Is the study design appropriate for the research question?

Yes

Is the rationale for, and objectives of, the study clearly described?

Yes

Are sufficient details of the methods provided to allow replication by others?

Yes

Are the datasets clearly presented in a useable and accessible format?

Yes

Reviewer Expertise:

dissemination and implementation science, cancer prevention, sexual health

I confirm that I have read this submission and believe that I have an appropriate level of expertise to confirm that it is of an acceptable scientific standard, however I have significant reservations, as outlined above.

# Reviewer report: Reviewer response for version 1

**DOI:** 10.21956/hrbopenres.14954.r34069

**Published:** 2023-06-13

**Authors:** Erika L. Crable

**Affiliations:** 1 University of California San Diego, San Diego, California, USA

The authors discuss important topics in dissemination and implementation strategy design – tailoring strategies and measuring the success of tailored strategies. They describe a funded program of research consisting of multiple projects to generate knowledge about implementation strategy tailoring. This is an exciting body of planned research. I have made a few questions/comments for the authors to consider, with the goal of enhancing clarity for readers.

Abstract: The authors note that “ *there has been less focus on the part of tailoring where…” *This is a bit about diction, but are activities where stakeholders prioritize and select strategies an intrinsic part of how to tailor or is it one approach to tailoring? I’d argue is it one approach (of many) since strategies could also be tailored based on data-driven approaches whereby stakeholder-provided data inform strategy tailoring, but stakeholders are not specifically involved in selecting strategies. This is a minor detail about phrasing that has major implications for how we conceptualize methodological approaches to tailoring.

Introduction: The authors describe several aims and research/training activities in the introduction. The first part of the introduction sets the reader up to expect a scoping review. Then aims to conduct a mixed methods study and multiple case study approach are described, followed by plans to enhance implementation science capacity with a training program. As a reader, I wanted to understand the purpose of summarizing this research program upfront, before hearing about all of its parts. You set this up well in the Abstract so perhaps that language could be used within the first paragraph of the introduction. It might be helpful to note that there is a need to identify core components of tailoring and measuring tailoring success, which has catalyzed the development of the CUSTOMISE research program, before introducing any of the bolded text about aims and activities. That initial framing could help the reader understand the relationship between the bolded statements throughout the introduction, and plainly see the importance of this novel program of research.

 Much of the proposed research is already underway and reported in more detail in other published protocols so providing detailed feedback here would be irrelevant. However, this again raises the question of ‘why report on this program of research when its individual components are already reported in protocol papers?’ I do think there is merit in doing so, but it would be helpful for the authors to spell this out for readers.

Session 1 - Determinant identification and initial prioritization: The authors note that they are using validated scales to assess inner setting measures per CFIR and readiness for implementation. However, no rationale for these scales or framework are offered. It would be useful to understand their rationale for selecting these particular scales and  framework, given the large repository of other scales and frameworks that exist and could have been chosen. How are the researchers operationalizing these scales and framework for their specific study?

Discussion: The authors describe their overarching aim as accelerating "the implementation of evidence into policy and practice". They have well-articulated how they expect tailored strategies will impact practitioners and practice, but little is said about how they plan to impact policy (a lofty goal which merits its own program of research). It would be helpful if they could articulate which policy/policies they hope to influence/create, at which level of policy (Big P, little p, national, local, etc.) they plan to impact, and their approach/strategies for doing this. There is a short discussion about how dissemination strategies will be “sensitive to” national policy, but that is unlikely to amount to the "implementation of evidence into policy". If a direct and intentional approach to translating evidence into policy is not part of the research program, I suggest the authors amend the opening sentence of the discussion to focus only on the practice changes they aim to achieve.

 Thank you for the opportunity to review this exciting body of research. I look forward to learning from this important work.

Is the study design appropriate for the research question?

Yes

Is the rationale for, and objectives of, the study clearly described?

Partly

Are sufficient details of the methods provided to allow replication by others?

Yes

Are the datasets clearly presented in a useable and accessible format?

Not applicable

Reviewer Expertise:

Dissemination and implementation science, health policy, dissemination strategy tailoring.

I confirm that I have read this submission and believe that I have an appropriate level of expertise to confirm that it is of an acceptable scientific standard.

# Reviewer report: Reviewer response for version 1

**DOI:** 10.21956/hrbopenres.14954.r33378

**Published:** 2023-01-30

**Authors:** Tracy Finch

**Affiliations:** 1 Department of Nursing, Midwifery & Health, Northumbria University, Newcastle upon Tyne, UK

This paper sets out an ambitious mixed methods programme of research to advance knowledge and application of tailored implementation as a key endeavour for Implementation Science. The case for advancing the conceptualisation, and operationalisation of implementation tailoring is clear and well made. The gaps in knowledge referred to, are recognisable to those already working in this area of research. Separate objectives are presented around updating existing knowledge (evidence synthesis) and developing new and more detailed knowledge of how tailoring is undertaken, and how it is experienced by those involved in the process.

 The multi-methods, multiple work streams research design presented includes research methods appropriately aligned to the different study objectives. These include evidence synthesis from reviews of conceptualisation and effectiveness of tailoring; evaluation of tailoring processes being undertaken in primary studies; multiple case studies of experiences of the tailoring process. For investigation of the tailoring process in detail, one primary study is identified at this point, where the researchers are presumably already involved and have access to the detailed data described for answering the questions set out in this programme protocol.

 The methods presented are clear, and as replicable as might be expected in a study programme protocol, with as much detail as is likely possible at time of publication. References are made to already published protocols for the evidence synthesis/review elements of the programme. In relation to one of the primary studies (implementation tailoring in relation to DAFNE), detailed aspects of all elements of the study design are provided. The quantitative data collection tools are mostly validated and/or published measures; topics for qualitative data collection are clearly described for this stage of the programme.

 For the multiple case study part of the design, some more detail on how additional case studies would be identified and recruited might be helpful: the tailoring process described for the DAFNE study is itself very comprehensive and in-depth, aligning to what might be considered ‘gold standard’ tailoring. How variable are the case studies, once identified, likely to be in terms of their approach and detail in the tailoring process? This is, however, a question that can be investigated as part of the programme itself. I do have some reservations that the detailed tailoring process described to be undertaken with participants in the DAFNE study (over 3 x 1hr workshops) can be feasibly completed in the time allocation described (to include, for example, ranking and prioritisation of up to ten strategies individually, discussing reasons etc), given that achieving consensus in teams on the different tasks may be more challenging than anticipated. It should be acknowledged however, that the tailoring process is set by the primary study itself (rather than the programme reported in this protocol), and assessment of feasibility of undertaking detailed implementation tailoring will contribute valuable knowledge to this research programme on implementation tailoring. I very much look forward to hearing the outcomes of this important research.

Is the study design appropriate for the research question?

Yes

Is the rationale for, and objectives of, the study clearly described?

Yes

Are sufficient details of the methods provided to allow replication by others?

Yes

Are the datasets clearly presented in a useable and accessible format?

Yes

Reviewer Expertise:

Implementation science in general; use of theory in implementation science; normalisation process theory; tailored implementation; qualitative process evaluation of complex interventions

I confirm that I have read this submission and believe that I have an appropriate level of expertise to confirm that it is of an acceptable scientific standard.
